# Taphonomy of the teleost *Tselfatia formosa* Arambourg, 1943 from Vallecillo, NE Mexico

**DOI:** 10.1371/journal.pone.0280797

**Published:** 2023-02-01

**Authors:** Eva Susanne Stinnesbeck, Fabian Herder, Jes Rust, Wolfgang Stinnesbeck

**Affiliations:** 1 Steinmann-Institut, Rheinische Friedrich-Wilhelms-Universität, Bonn, Germany; 2 Zoologisches Forschungsmuseum Alexander Koenig, Bonn, Germany; 3 Institut für Geowissenschaften, Ruprecht-Karls-Universität, Heidelberg, Germany; University of Florida, UNITED STATES

## Abstract

The platy limestone deposit of Vallecillo in northeastern Mexico is dated to the early-middle Turonian (Late Cretaceous) and known to contain a variety of well-preserved vertebrate fossils. One of the most common fish species is the teleost *Tselfatia formosa*. A review of 149 individuals reveals the presence of two types of body shapes (diamond-shaped and torpedo-shaped individuals) which is interpreted as sexual shape dimorphism (SSD). A unimodal size distribution illustrates a dominance of diamond-shaped specimens, but both body shape types are present in small (young) and big sized (old) individuals. The abundance of well-articulated and complete specimens suggests that *T*. *formosa* populated deep levels of the water column, which excluded buoyancy and flotation as well as carcass disintegration near the surface. The reconstruction of the dorsal and anal fins suggests the presence of a membrane between each fin ray and allows for ecological comparison of *T*. *formosa* with modern fan fishes.

## 1 Introduction

The teleost *Tselfatia formosa* was originally described by Arambourg [1, 2] from the Upper Cenomanian of Jebel Tselfat (Morocco) and is also known from the Upper Cenomanian of Cinto Eugeano in Italy [3], the Cenomanian-Turonian of Texas and Croatia [4], the Upper Cenomanian or Lower Turonian of Misburg in Germany [5], and the uppermost Cenomanian- middle Turonian Vallecillo locality in Mexico [6]. *T*. *formosa* is therefore presently restricted to the Cenomanian and Turonian. Individuals from the Austin Chalk (Coniacian-Santonian age) of Texas referred to *T*. *formosa* by Bardack and Teller-Marshall [4], differ from this taxon by lacking laterally keeled caudal vertebrae; they were assigned by Maisch und Lehmann [5] as *Tselfatia* sp. *T*. *dalmatia*, originally described from the Cenomanian-Turonian of Yugoslavia by Bardack and Teller-Marshall [4], is distinguished from *T*. *formosa* by a deeper and shorter body and a raised position of the pectoral fin in regard to the vertebral column [4, 5].

The systematic position of *Tselfatia formosa* was discussed by numerous authors. Taverne [7] proposed a close relationship to Bananogmiidae, instead of Beloniformes, Scombroidei or Osteoglossomorpha as previous suggested by Arambourg [1, 2] and Patterson [8], among others [4, 5]. The taxon is presently assigned to the order Tselfatiiformes (Bananogmiiformes) which is split into the Eoplethodidae, Plethodidae and Protobramidae. *Tselfatia* is placed within Plethodidae and shares the characteristic segmented fulcra of the first anal and dorsal fin rays with *Dixonanogmius* [9, 10].

**Fig 1:**
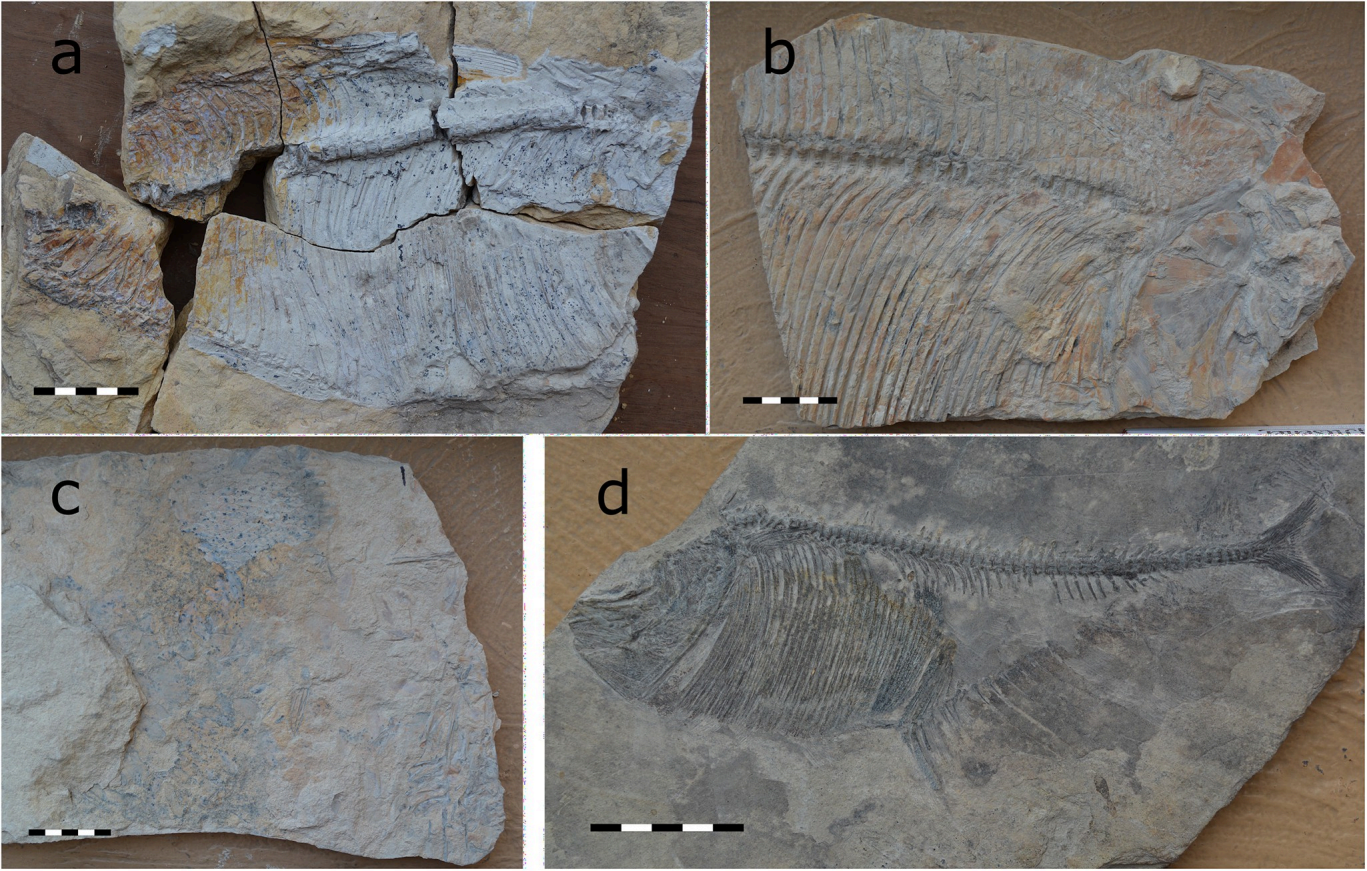
*Dixonanogmius* sp. from the Middle-Late Santonian Los Temporales locality (CPC-2837) (A) and from the Cenomanian San Carlos quarry (CPC-2838 and CPC-2836) (B and C). *T*. *formosa* from the Turonian Mesa Las Tablas quarry in southern Coahuila (CPC-2835) (D). All scales 50 mm.

In Mexico fossil tselfatiiform fishes are represented by *Bananogmius*, *Dixonanogmius* and *Tselfatia*. *Bananogmius* was mentioned by González-Rodríguez et al. [11] to exist in the Albian Tlayúa locality in Puebla but has not yet been officially described. A similar fish to *Bananogmius* was reported by Applegate [12] from the quarries near Tepexi de Rodríguez in Puebla and a single specimen of *Bananogmius* was documented by Giersch [9] from the Turonian La Mula quarry in northern Coahuila but remains unpublished. We additionally suggest that *Dixonanogmius* is present in the Middle-Late Santonian Los Temporales locality (Fig 1A) and in the Cenomanian San Carlos quarry (Fig 1B and 1C). In addition to the abundant material from Vallecillo, *T*. *formosa* is here also documented from the Turonian Mesa las Tablas quarry in southern Coahuila (Fig 1D) and was already reported by Alvarado-Ortega et al. [13] from the Turonian Huehuetla quarry. *Tselfatia* sp. has been recorded from La Mula (Cenomanian-Turonian; Coahuila), Xilitla (Upper Turonian; San Luis Potosí) and the Turonian Arroyo las Bocas locality [14–16].

*Tselfatia formosa* from Vallecillo was first described by Blanco-Piñón [6]who identified various characters to characterize this species. These include a skull significantly higher than long, 65 vertebrae forming the vertebral column, small pectoral fins ventral to the vertebral column, a dorsal fin with 40 rays, an anal fin with 20 rays, a segmentation by pseudo-fulcra and lepidotrichia on the 5^th^ ray of the dorsal and the 3^rd^ ray of the anal fin, and fused hypurals forming a fan-like structure [4, 7, 6]). The specimens presented here confirm this interpretation.

## 2 Materials and methods

The fossil material of *Tselfatia formosa* analyzed here consists of 149 individuals from Vallecillo. The collection is based on museum material and was not collected randomly from the surface of the outcrop. Fossils are housed in the Colección Paleontológica de Coahuila in the Museo del Desierto at Saltillo and in the collection of Ing. Mauricio Fernández Garza in Monterrey. The latter material will form part of a new paleontological museum in Monterrey dedicated to the Vallecillo fossil lagerstätte. This museum is currently under construction and will be opened 2023. The material of Mr. Fernández Garza will be housed in the museum of Saltillo until the new museum opens. All specimens received a catalog number of the museum of Saltillo (CPC) and in some cases from the Instituto Nacional de Antropología y Historia (REG). No permits were required for the described study, which complied with all relevant regulations.

Fragmentary specimens devoid of essential body compartments were not used for size range documentation and body shape analysis but were still used to obtain information about e.g. the dorsal or anal fins, the mouth, or the preservation of the vertebral column. The total length and the body depth of individuals was measured following the descriptions of Hastings et al. [17].

**Articulation and completeness** of *T*. *formosa* are differentiated allowing for the presence of highly disarticulated individuals that are osteologically complete. There are five groups identified in both fields, completeness and articulation. We distinguished complete (c), nearly complete (nc), partially complete (pc), mostly incomplete (mic) and incomplete (in) specimens. *Complete individuals* may lack only small osteological details such as single fins or ribs. Nearly complete individuals lack up to two fins, ribs and fin rays, but the body remains mostly complete. *Partially complete specimens* lack essential body parts such as the caudal fin, vertebrae, fins and ribs. To about half of the specimen is lost in this group. *Mostly incomplete specimens* are devoid of >50% of essential body parts. Specimens comprising only one body compartment (e.g. only the skull is present) are here considered as *incomplete*. The following five groups are differentiated regarding articulation: articulated (a), nearly articulated (na), partially articulated, mostly disarticulated (md) and disarticulated (d), as already discussed by Stinnesbeck et al. [18].

**Fig 2:**
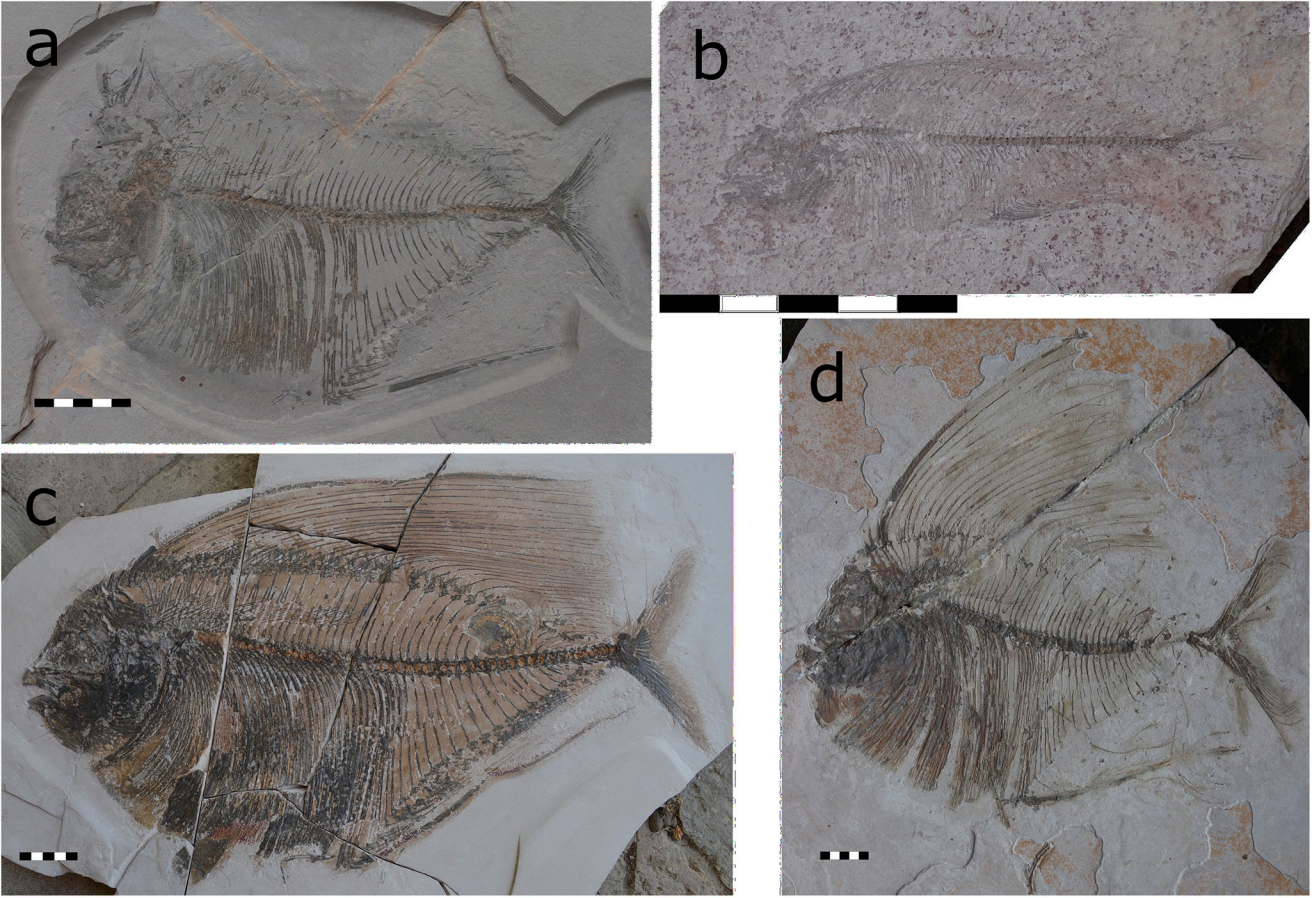
Preservational stages of the dorsal fin of *T*. *formosa*. Disarticulated dorsal fin (CPC-2892) (A). Folded dorsal fin classified as ‘not spread’ (ns) within a range of 0°—20° (CPC-2841) (B). Middle spread dorsal fin (ms) within 21° to 40° (CPC-2948; REG2544 PF48) (C) and full spread (fs) dorsal fin (>41°) (CPC-2839) (d). All scales 50 mm.

The preservation of **unpaired fins** is here classified into four groups: disarticulated (d), not spread (ns), spread to some extend (middle spread: ms) and fully spread (fs). Specimens with disarticulated dorsal or anal fin rays are classified as disarticulated (Fig 2A). Dorsal and anal fins which are retracted remain parallel to the main body reaching an angle of between 0°—20° (Fig 2B). A middle spread dorsal or anal fin is here interpreted to range between 21°—40° (Fig 2C). Fully erected fins reach to 90° (>41°) (Fig 2D).

Highly disarticulated or incomplete specimens were not used for the determination of size range nor the analysis of body shape. Individuals used for the determination of size range were also used for body shape analysis and for the correlation of size and shape.

There are five categories for **column breaks**: disarticulation (d), no breaks (nb), break of column in the anterior region (head region: hr), break in the posterior region (tail region: tr) and break in both anterior and posterior region (head and tail region: htr). The anterior region of the body is defined as the distance from the most posterior point of the operculum to the first ray of the anal fin. Vertebrae located beneath the operculum are counted to the head region. The posterior region marks the distance from the first anal fin to the base of the caudal fin. Multiple column breaks anterior and posterior of the body are counted to the last category (head and tail region).

There are three positions identified for the **mouth**. Disarticulated (d), closed (c) and open (o) (> 0°). Specimens with a disarticulated skull are counted as disarticulated.

**Fig 3:**
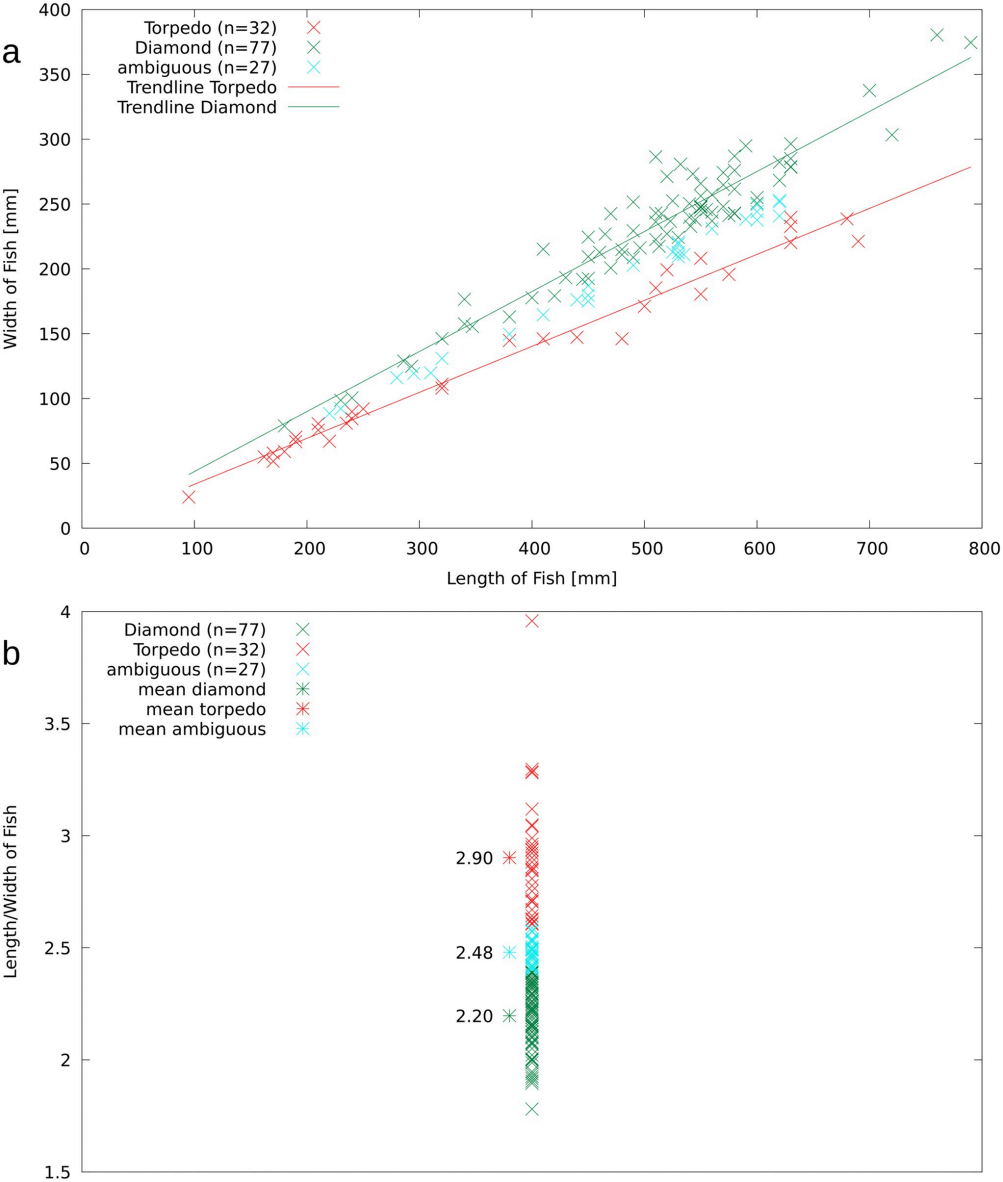
Body length of *T*. *formosa* in relation to body depth (A). Diamond-shaped individuals are deeply keeled and twice as long as wide, in contrast to torpedo-shaped individuals that are three times longer than wide with a more streamlined body outline (B).

**Fig 4:**
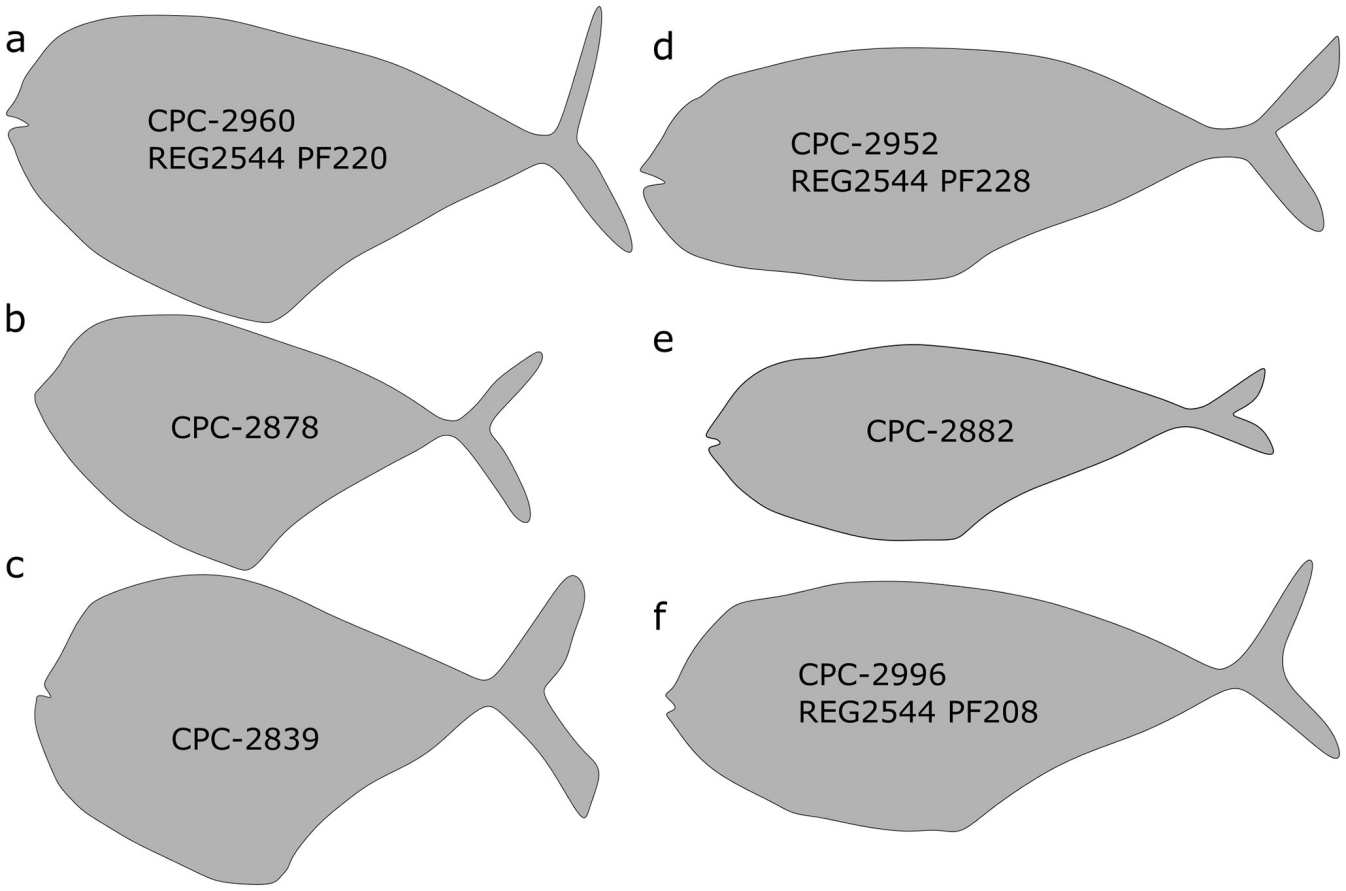
Body shape types documented in *T*. *formosa*. Diamond-shaped individuals are deeply keeled (A—C) while torpedo-shaped individuals show a streamlined body outline (D—F). The specimen collection numbers are placed inside each shape.

The **body shape** of *T*. *formosa* is differentiated into two groups: diamond-shaped individuals (diamond) and torpedo-shaped specimens (torpedo), while disarticulated (d) and ambiguous individuals are also present. Diamond-shaped specimens are twice as long as wide, with a length to width body ratio of 2:1 (Fig 3A and 3B). Individuals show a curved back with a front higher than the tail. The attachment point of the fifth segmented ray at the skull forms the highest point of the back. Specimens show a notable “nook” between the pelvic and anal fins, which creates the deepest point of the ventral body and a rhombic shape (Fig 4A–4C). Torpedo-shaped individuals are three times longer than wide and thus present a length to width body ratio of 3:1 (Fig 3A and 3B). They are dorsally less curved when compared to diamond-shaped individuals, while the deeply keeled belly seen in diamond-shaped specimens is either absent, or faintly marked. The highest dorsal point is reached in the middle of the arched back and the deepest point before the anal fin (Fig 4D–4F). There are no other features identified here to distinguish the two body shapes and thus no traits that would justify the erection of two different *Tselfatia* species. Specimens described as ambiguous are not clearly differentiated as diamond or torpedo-shaped. These individuals are not keeled enough to distinguish a rhombic shape and not long enough to be interpreted as torpedo-shaped (Fig 3A and 3B).

## 3 Results

### 3.1 Body shape

**Fig 5:**
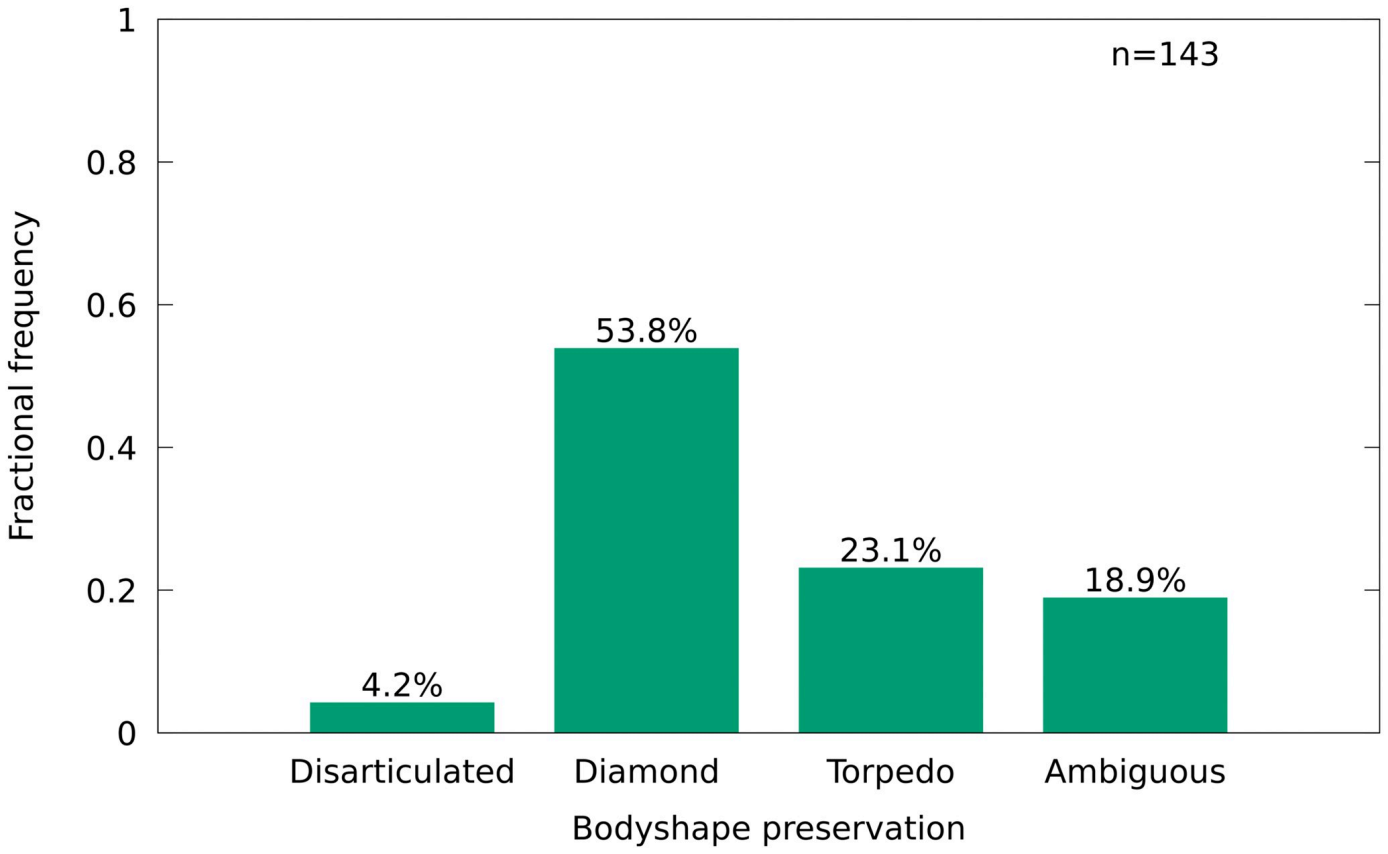
Diamond-shaped, torpedo-shaped and disarticulated individuals of *T*. *formosa* in percentage.

53.8% of the individuals are here referred to the diamond-shaped type, whereas 23.1% are classified as torpedo-shaped (Fig 5). 4.2% of the individuals were disarticulated, thus not allowing for a precise classification of body shape and 18.9% are interpreted as ambiguous.

### 3.2 Size range and body shape

**Fig 6:**
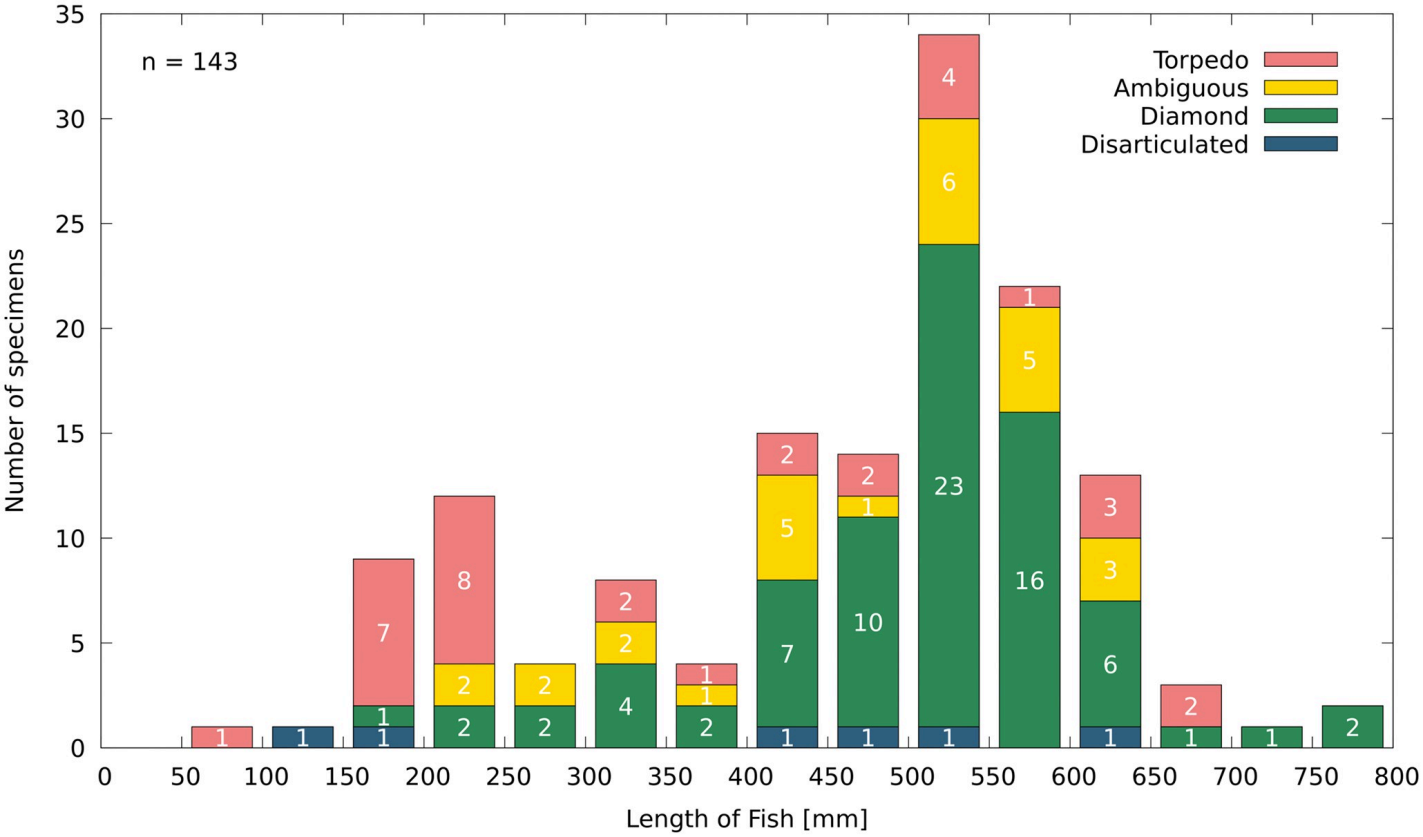
Size range distribution of 143 individuals of *T*. *formosa* from Vallecillo correlated to both body shape types. Torpedo-shaped individuals are marked in red and diamond-shaped specimens in green. Disarticulated individuals are presented in blue and ambiguous individuals in yellow.

The size range distribution for *T*. *formosa* is illustrated based on the total body size of 143 specimens from the platy limestone deposit of Vallecillo (Fig 6). It should be noted that the material was collected for museum purposes and is thus selective. It was not collected randomly from the surface of the outcrop, but nevertheless no preference was given to large specimens as abundant small individuals are also present. The smallest specimen reaches a length of 95 mm, while the largest is 790 mm long. Small individuals below 50 mm are not present, most likely as a result of diagenetic destruction or lack of preservation. A similar preservational bias has been described for *N*. *gutturosum* and *G*. *roberti* from Vallecillo lacking individuals smaller than 50 mm [18, 19]. A unimodal size distribution is illustrated in Fig 6 by an abundance peak of individuals between 500 to 600 mm length. According to these data, the former Vallecillo ocean was therefore predominantly populated by individuals ranging from 400 to 650 mm length, which are here interpreted as adults. Juvenile individuals ranging from 150 to 400 mm length are less abundant. Specimens ranging from 50–150 mm, 250–400 and 650 to 800 mm length are rare.

Regarding the two types of body shape, small individuals ranging from 150 to 250 mm length are frequently torpedo-shaped, whereas adult specimens of 450 to 600 mm length are majorly diamond-shaped. Nonetheless, both diamond or torpedo shaped specimens are identified in juveniles and adult individuals in the present material (Fig 6). Ambiguous individuals are majorly identified in the range of 500 to 600 mm length.

### 3.3 Taphonomy and preservation

#### 3.3.1 Gut content

Gut content is preserved as compact recrystallized mass without identifiable matter, similar to the situation identified in *Goulmimichthys roberti* from Vallecillo [18]. This amorphous mass is often elliptical in outline, allowing for a distinction of the cloacal canal between the pelvic and anal fins. Nonetheless, stomach content is rare (20.2%) and a majority of specimens (79.0%) lacks intestinal remains.

#### 3.3.2 Scales and color

**Fig 7:**
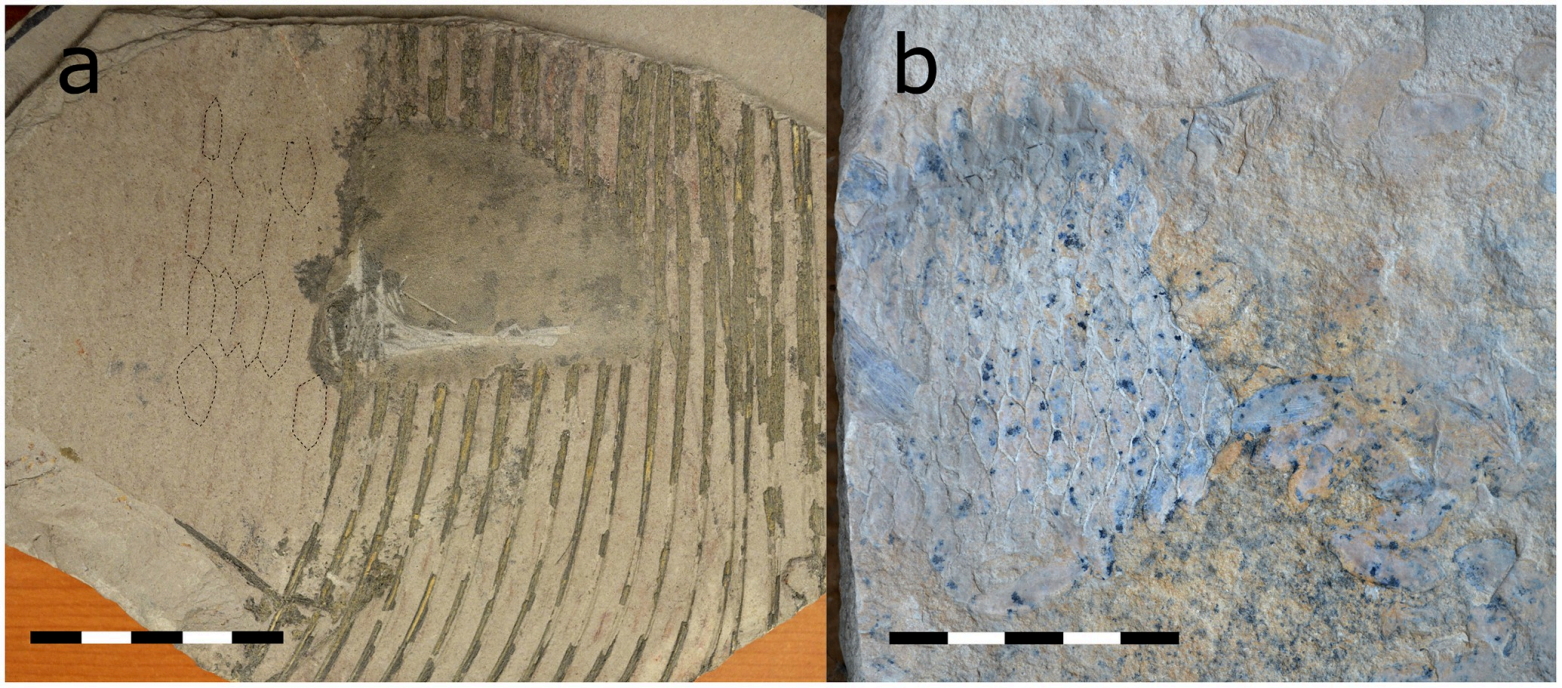
Scales of *T*. *formosa* from Vallecillo (CPC-2824) (A) in comparison to *Dixonanogmius* sp. from San Carlos (CPC-2836) (B). All scales 50 mm.

Scales are rhombic in shape and preserved as shadow imprints in three *T*. *formosa* specimens from Vallecillo (Fig 7A). One specimen interpreted as *Dixonanogmius* sp. from the San Carlos locality (Cenomanian) in northern Coahuila shows a similar shape of scales as identified here in *Tselfatia formosa* from Vallecillo (Fig 7B). Horizontal color patterns forming stripes as described by Giersch [9] were not observed in the present material of *T*. *formosa*.

#### 3.3.3 Completeness and articulation

**Fig 8:**
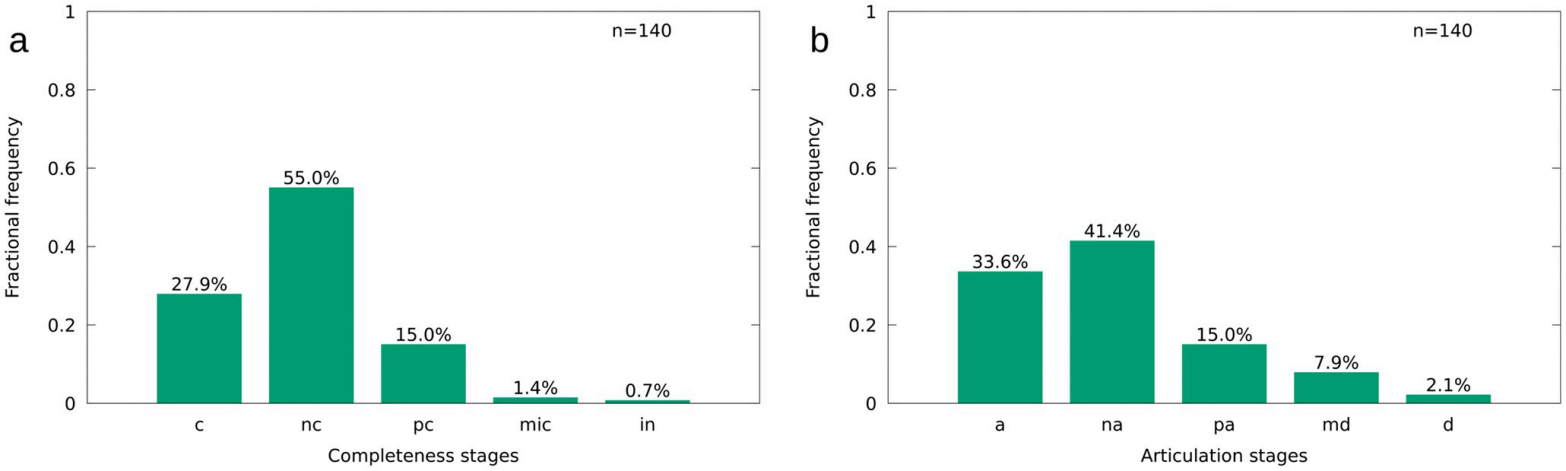
Completeness (A) and articulation (B) of *T*. *formosa* from Vallecillo in percent of the total number of individuals. Completeness stages differentiated here (Fig. 8A) include complete (c), nearly complete (nc), partially complete (pc), mostly incomplete (mic) and incomplete (in) specimens. Articulation stages differentiated (Fig. 8B) include articulated (a), nearly articulated (na), partially articulated (pa), mostly disarticulated (md) and disarticulated (d) individuals.

All specimens of *T*. *formosa* presented here are laterally preserved. Most individuals are nearly complete (55.0%) or complete (27.9%) (Fig 8A), while 15.0% are partially complete. Only 1.4% of the individuals are mostly incomplete and 0.7% are incomplete.

The majority of the individuals are nearly articulated (41.4%) and 33.6% are articulated (Fig 8B), while 15.0% are partially articulated and 7.9% classified as mostly disarticulated. 2.1% of the specimens are disarticulated. Individuals are therefore mostly complete rather than articulated, and generally well preserved (disarticulation and incompleteness is rare). Even though, it needs to be emphasized that the collection analyzed here is biased, as mainly well preserved specimens have been collected for exhibition purposes.

#### 3.3.4 Fin preservation

**Fig 9:**
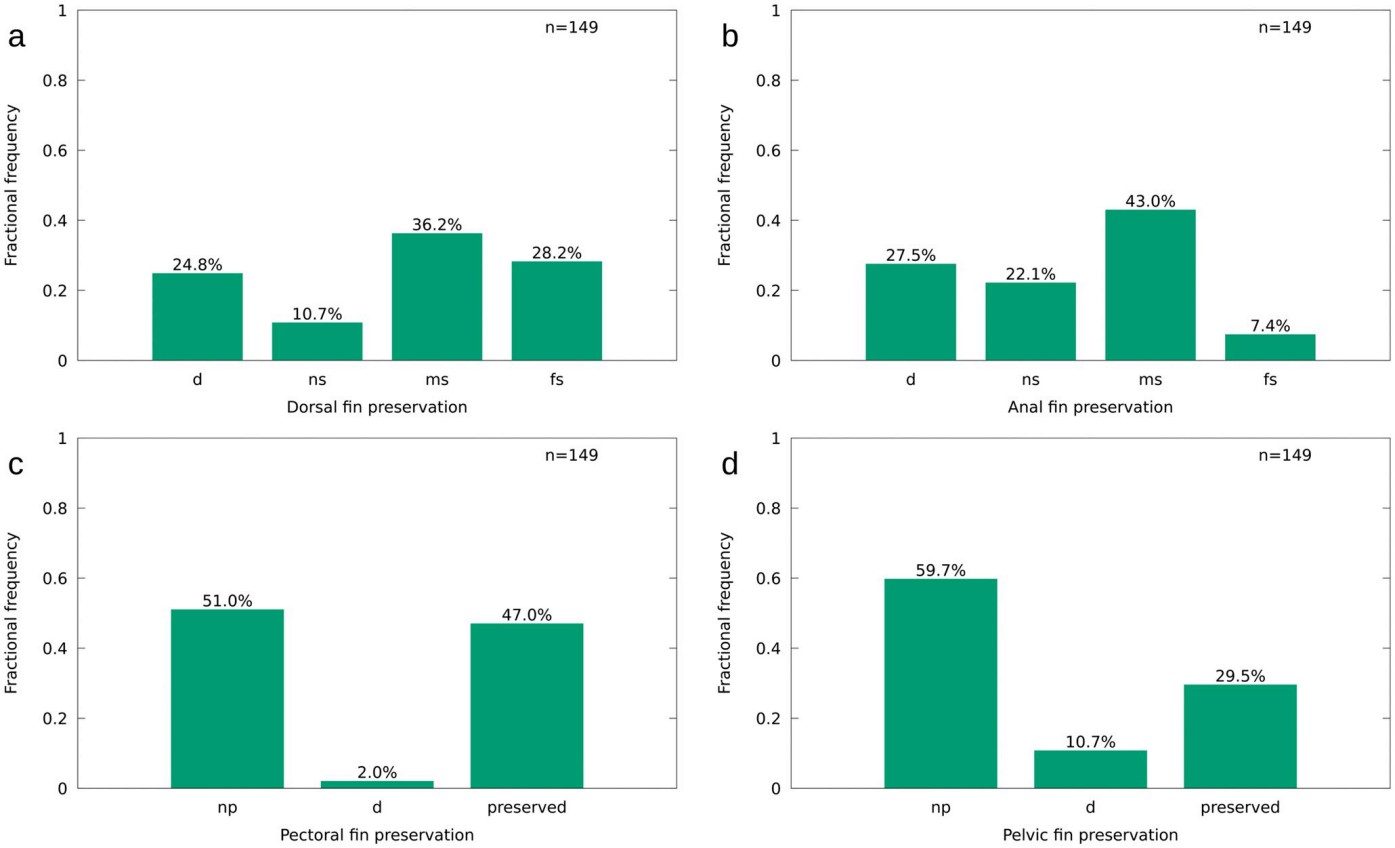
Preservation of the dorsal (A), anal (B), pectoral (C) and pelvic fins (D) of *T*. *formosa*, in percent of the total number of individuals. Disarticulated (d), not spread (ns), mostly spread (ms), fully spread (fs), not preserved (np) and preserved.

The dorsal fin of *T*. *formosa* is frequently preserved in a middle spread position (36.2%). A fully spread position of the dorsal fin is visible in 28.2%. In 24.8% of the specimens the dorsal fin was disarticulated and a minority of 10.7% lack any fin spreading (Fig 9A).

The anal fin is mostly spread in a middle position (43.0%). 27.5% possessed a disarticulated anal fin and 22.1% lacked fin spreading. Only 7.4% showed a full erection of the anal fin (Fig 9B). In conclusion, the dorsal fin is more frequently erected in the present material than the anal fin.

Pectoral fins are preserved in 47.0% of the specimens, but a majority of 51.0% lacks pectoral fins. In only 2.0% of the individuals the pectoral fin is disarticulated (Fig 9C).

In the majority of individuals the pelvic fins are not identified (59.7%). In 29.5% of the specimens the pelvic fins are preserved but 10.7% possess disarticulated pelvic fins (Fig 9D). Pectoral fins are better preserved than pelvic fins. It has not been possible to calculate the percentage of pectoral and pelvic fin spreading due to bad preservation.

#### 3.3.5 Preservation of the vertebral column

**Fig 10:**
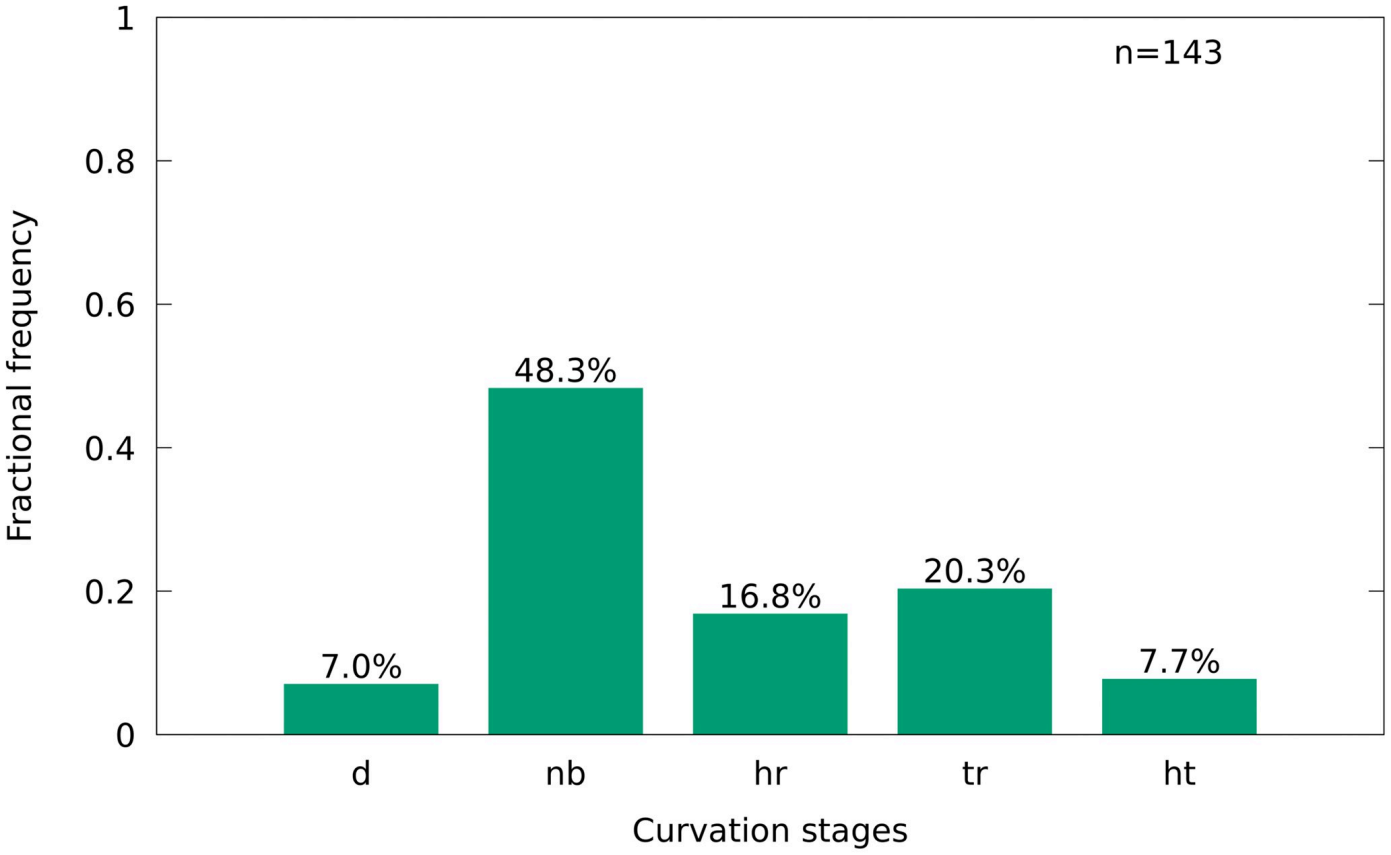
Preservation of the vertebral column in *T*. *formosa*, in percent of the total number. Disarticulated (d), no break (nb), break in head region (hr), break in tail region (tr), break in head and tail region (ht).

The vertebral column is mostly straight, different to the frequent curvature identified in specimens of *Goulmimichthys roberti* from Vallecillo [18]. In 48.3% of the individuals the vertebral column is unbroken. In 20.3% the column is broken in the posterior region and in 16.78% in the anterior region, while 7.7% show fractures in both regions. In 7.0% of the specimens the vertebral column is disarticulated (Fig 10).

A break of the vertebral column is therefore identified in about 50% of the specimens but it is not particularly frequent in specific positions of the column. This differs from *G*. *roberti* from Vallecillo where breakage of the column was abundant in the tail region [18].

#### 3.3.6 Reconstruction of the dorsal and anal fins

**Fig 11:**
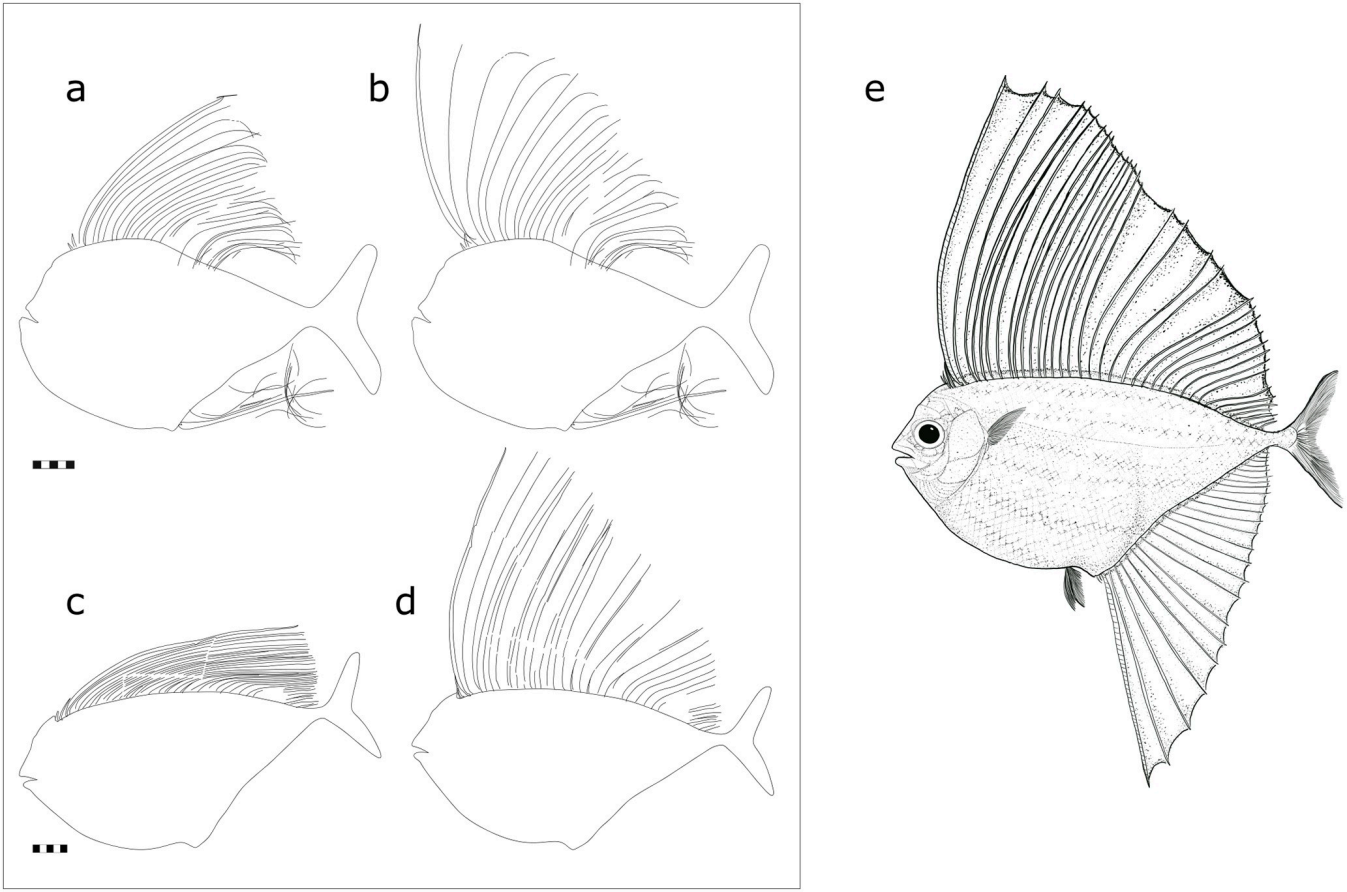
Reconstruction of the dorsal fin of *T*. *formosa*. Fossil outline as identified in CPC-2839 and CPC-2948; REG2544 PF48 with semi-erected dorsal fin (A and C) in comparison to its modification (B and D) with erected dorsal fin. Schematic reconstruction of the dorsal and anal fin including a membrane between the fin rays (E) and scale color as suggested by Giersch (2014). Both scales 50 mm.

The major fin ray of both the anal and dorsal fin is exceedingly long, reaching the base of the caudal fin when retracted. The dorsal and anal fin rays of *T*. *formosa* are therefore much longer than described by Taverne [7] and Giersch [9]. To obtain a new reconstruction of both fins, we drew the contour of majorly complete fossils (Fig 11A and 11C) and rotated each fin ray at the basis to obtain a vertical erection of the fin (Fig 11B and 11D). This allows for a reconstruction of the original dimension of the dorsal and anal fins, which coincides with incomplete specimens of *T*. *formosa* (Fig 11E).

## 4 Discussion

### 4.1 Size range

The abundant material of *Tselfatia formosa* from Vallecillo ranges from 50 to 800 mm length. A unimodal distribution is identified similar to that found for *G*. *roberti* [18] from the locality. However, in contrast to *G*. *roberti* the highest abundance is here registered for individuals ranging between 500 and 600 mm length (Fig 6). These adult individuals, with total lengths of 400 to 650 mm, are notably more abundant than juvenile specimens ranging between 150 to 350 mm length. Specimens ranging from 50 to 150 mm, 250 to 300, 350 to 400 and 650 to 800 mm length, are underrepresented. The specimens presented here (n = 143) significantly extend both, the number of individuals of *T*. *formosa* recorded to date from Vallecillo and their size range (e.g. [6, 9, 20, 21]). Individuals recorded by Blanco [6] range from 160 to 610 mm length. Smaller and bigger-sized individuals were recorded by Ifrim [20] and Giersch [9]. Both authors presented a unimodal size range based on small specimen numbers. In general the size range presented here coincides with data presented by these latter authors, except for specimens between 180 and 280 mm length missing in the size range of Ifrim [20]. The material presented here is based on museum collections and was not collected randomly. The size range is nevertheless similar to that presented by Ifrim [20] which is based on randomly collected material. We therefore consider our results as unbiased. The total set of data indicates that the former Vallecillo ocean was majorly inhabited by adult individuals (>400 mm length). The abundance of big-sized specimens can also be interpreted to indicate high adult mortality. Seasonal migration of small specimens, as seen in *N*. *gutturosum* from Vallecillo [19], appears unlikely for *T*. *formosa*, due to the low number of small-sized individuals here interpreted as juveniles. These did apparently not persist in the Vallecillo region, which represents an open marine environment with water depths of about 200m [20, 22]. Rather, these juveniles may have populated shallow areas near the coast, although to date there is no positive evidence for this hypothesis. Future findings of small *T*. *formosa* in northern Coahuila (e.g. north of Múzquiz) could clarify this interpretation, as this region was placed in more shallow environments.

### 4.2 Body shape and sex

A torpedo-shaped and a diamond-shaped body type are here identified for *T*. *formosa* (Fig 4). There are no other osteological differences between the two forms, which are identified in both small and large-sized individuals (Fig 6). Torpedo shaped individuals show a length to width body ratio of 3:1, whereas in diamond shaped individuals the ratio is 2:1. Diamond-shaped individuals are more frequent in the length range of 500 to 600 mm, while torpedo-shaped specimens are abundant in smaller-sized individuals, ranging from 150 to 300 mm length (Fig 6). No study is known to us documenting these distinctly different body outlines in *T*. *formosa*, but an increase of body depth relative to the standard length was proposed by Patterson [8] to occur during the ontogeny of *Tselfatia*. For *T*. *dalmatica*, nevertheless, Maisch and Lehmann [5] argued that the differences in body proportions cannot be explained as an ontogenetic variation. Unfortunately, the authors provide no explanation for this conclusion nor do they discuss this in detail. However, if changes in body shapes were present during the ontogeny of *T*. *formosa*, one would expect a certain shape to be correlated to a certain size. Juvenile *Tselfatia* would then display a torpedo-like body shape during early ontogeny, changing into a diamond-like form when the adult stage is reached, or vice versa. Clearly, this is not the case in the Vallecillo material, as both torpedo- and diamond-shaped animals are represented by juvenile and adult specimens (Fig 3A). We therefore propose that the two body shape types rather represent sexual dimorphism, as previously identified in *N*. *gutturosum* from Vallecillo [19]. Such differences in male and female appearance are scarce in the fossil record but well studied in extant fishes [23–29]. Sexual shape dimorphism (SSD) can reflect e.g. locomotion, foraging, mating behavior or habitat requirements [30–32]. A slender shape is frequently correlated to habitats in which greater swimming activities are needed [33], consequently leading to shape differences depending on the complexity of the habitat. Differing body shapes of *T*. *formosa* could therefore be the result of different habitat preferences within the water column of Vallecillo. *T*. *formosa* living in higher energetic upper stages of the water column could then display a more slender body shape, while individuals populating the low energy deeper water developed a diamond-shaped form. Nevertheless, this interpretation is opposed by the absence of preservational differences between diamond- and torpedo-shaped individuals, as articulation and completeness are similar in both groups. In animals populating shallow depths of the ocean, disarticulation of carcasses is a common taphonomic feature as these individuals are exposed to extended decay during drifting and floating [34]. Animals which lived in deeper zones of the water column or at the sea floor are, on the other hand, often preserved complete and articulated. Therefore, no water column preferences (agitated and quiet water conditions) are associated to one or the other body shape and both types must have populated deep water levels. Furthermore it has been suggested that fishes with deep bodies generally associate with complex environments requiring for high maneuverability [33, 27]. This is arguably not the case for diamond-shaped *T*. *formosa*, which shared the same open marine environment populated by torpedo-shaped individuals. More likely, the differing body outlines may relate to foraging or reproductive reasons. An increase of the abdominal cavity is required in fishes for the accommodation of female reproductive organs; it is then associated with higher fecundity [27, 31, 35]. This adaptation may be present in diamond shaped individuals to maximize reproductive success and improve larval survival. The strategy has been documented for extant Carangidae, e.g. *Decapterus macrosoma* [36]. Females of *D*. *macrosoma* exhibit a deeper body depth, broader belly, bigger head and wider caudal fins than males of this species, which are narrow-bodied with smaller heads and wider dorsal and caudal fins [36]. Females store energy to facilitate the production of offspring, while males present adaptations to succeed in male to male competition [31, 36]. Furthermore, females of *D*. *macrosoma* exhibit bigger heads to maximize buccal volume and suction velocity. This is needed to acquire additional food to produce the eggs [36]. This latter adaptation is not identified in *T*. *formosa* due to insufficient skull preservation but nevertheless, gut contents are majorly seen in diamond-shaped individuals. This observation favors the assumption that stomach content is more common in diamond-shaped individuals, as these potential females needed to swallow additional quantities of food, which is therefore preserved more often. Based on these observations we tentatively interpret diamond-shaped individuals as females and more slender specimens as males.

Ambiguous specimens are not interpreted as a transitional form between diamond and torpedo-shaped individuals. A shift from one shape to the other caused by e.g. ontogenetic variation is here excluded. Ambiguous individuals are rather interpreted as a variation within *T*. *formosa*. These individuals are not precisely assigned to either the diamond or the torpedo form.

### 4.3 Taphonomy

**Fig 12:**
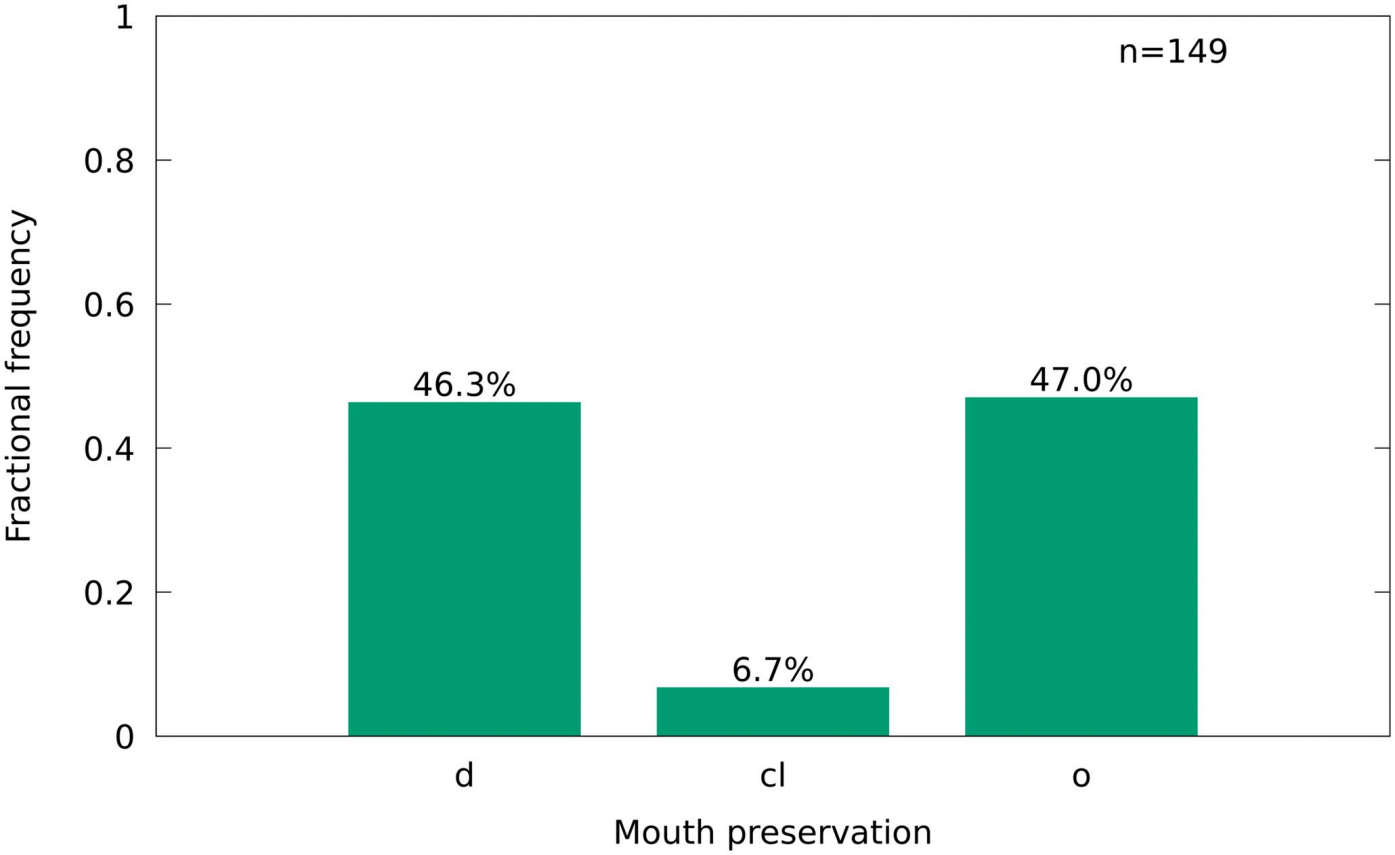
Mouth preservation of *T*. *formosa*, in percent of the total number. Disarticulated (d), closed (cl) and open (o).

*T*. *formosa* from Vallecillo is frequently well articulated and complete (Fig 8A and 8B). Dorsal and anal fins are often better preserved than the pectoral and pelvic fins. The dorsal fin is majorly erected in a middle-spread position (36.2%), or fully spread (28.2%), whereas the anal fin is majorly middle-spread (43.0%) or disarticulated (27.5%). The vertebral column is frequently articulated and breakage is rare (48.3% without break), without an indication of substantial weakness at the tail (posterior) or head (anterior) region. The mouth is frequently disarticulated (46.3%), but 47.0% of the specimens are documented with an open mouth (Fig 12). *T*. *formosa* is therefore regularly seen with spread fins and an open mouth, similar to *G*. *roberti* from Vallecillo [18].

**Fig 13:**
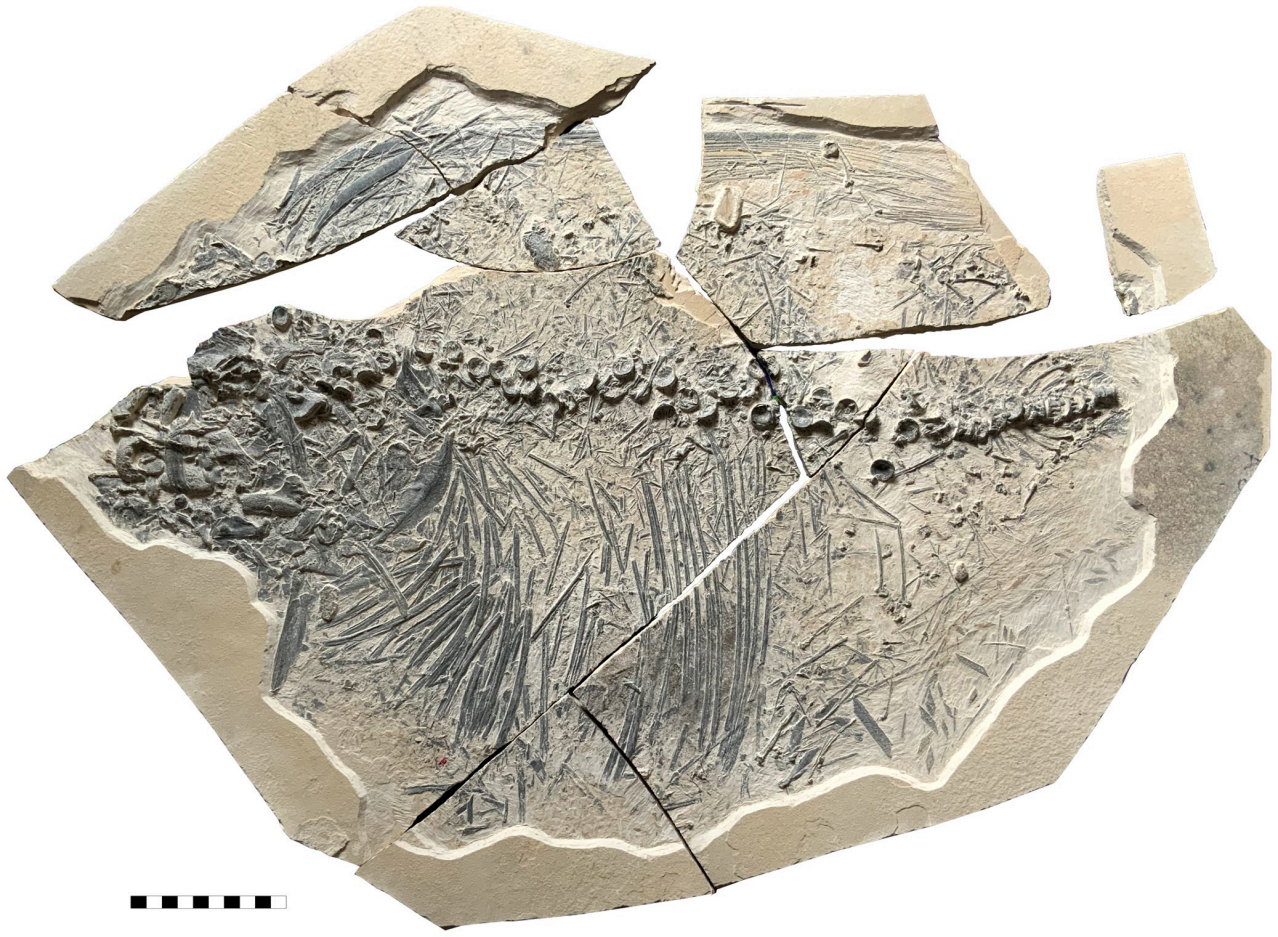
Disarticulated *T*. *formosa* (CPC-2833). The carcass disintegrated on the seafloor without bone dispersal caused by scavengers or currents. A phase of flotation is excluded. Scale 100 mm.

Due to the abundance of well-articulated and complete individuals we propose that no transport or flotation phase of the carcasses existed and that arrival on the sea bottom was rapid. Fishes were rapidly buried, thereby preventing substantial decay. Even in the few disarticulated individuals, displaced bones generally remained close to the main body and did not drift away (Fig 13). Fin rays usually split in two halves resulting in two major fin rays lying next to each other. In highly disarticulated specimens the pseudo-fulcra of the major fin ray split into small parts similar to box knife blades. Disarticulation caused by scavengers is not documented from Vallecillo, and bone dispersal by currents is extremely rare. The excellent preservation of *T*. *formosa* thus points to a lifestyle in deeper water zones of the former Vallecillo ocean. This interpretation agrees with assumptions by Ifrim [20] and Diedrich [37], the latter associating medium sized *T*. *formosa* with cold water fish.

### 4.4 Function of the dorsal and anal fin

**Fig 14:**
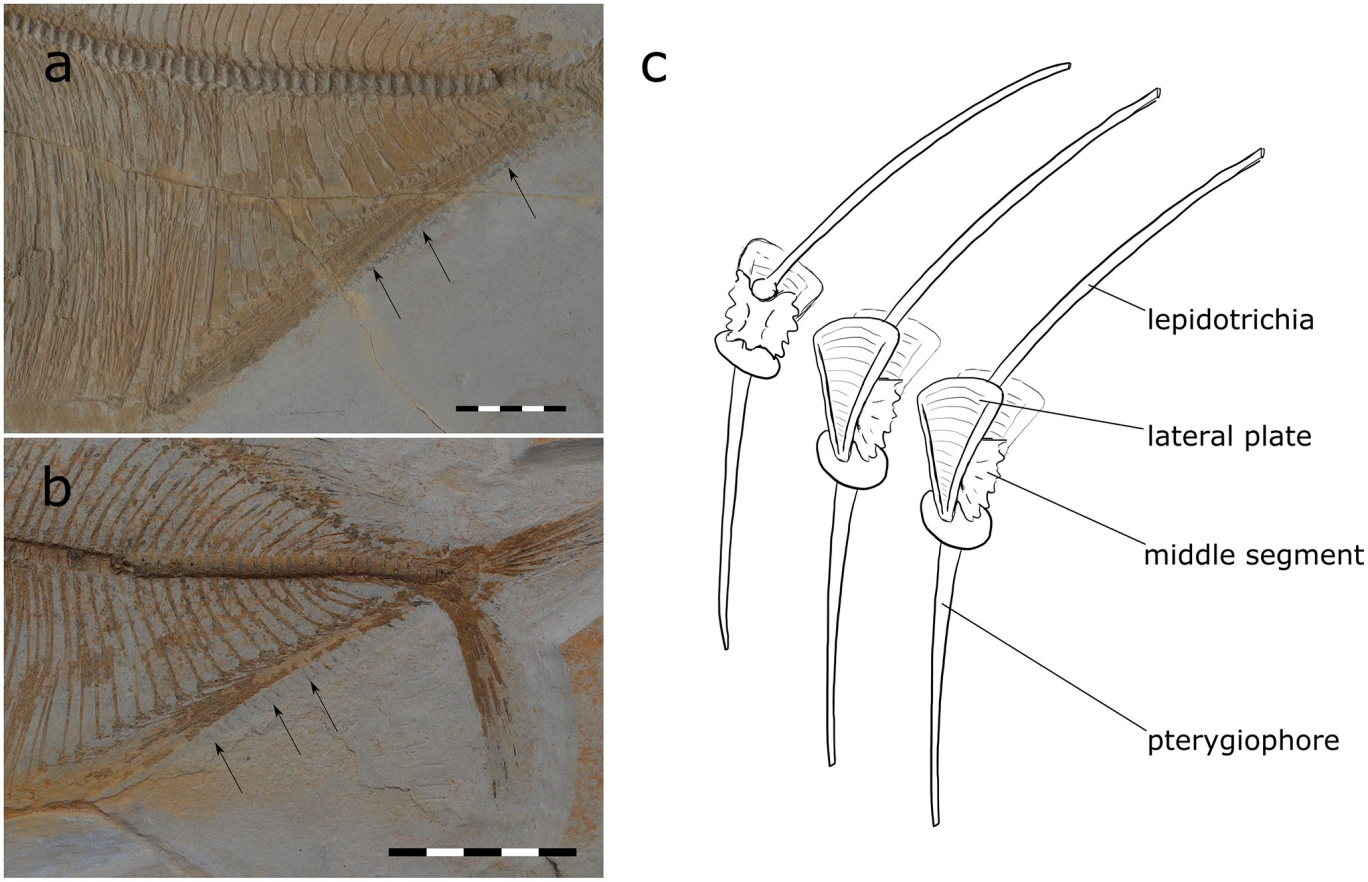
Retraction of the unpaired fins of *T*. *formosa* behind lateral plates (A and B) (CPC-2856 and CPC-2885). Reconstruction of the skeletal elements found in the unpaired fins (C). All scales 50 mm.

**Fig 15:**
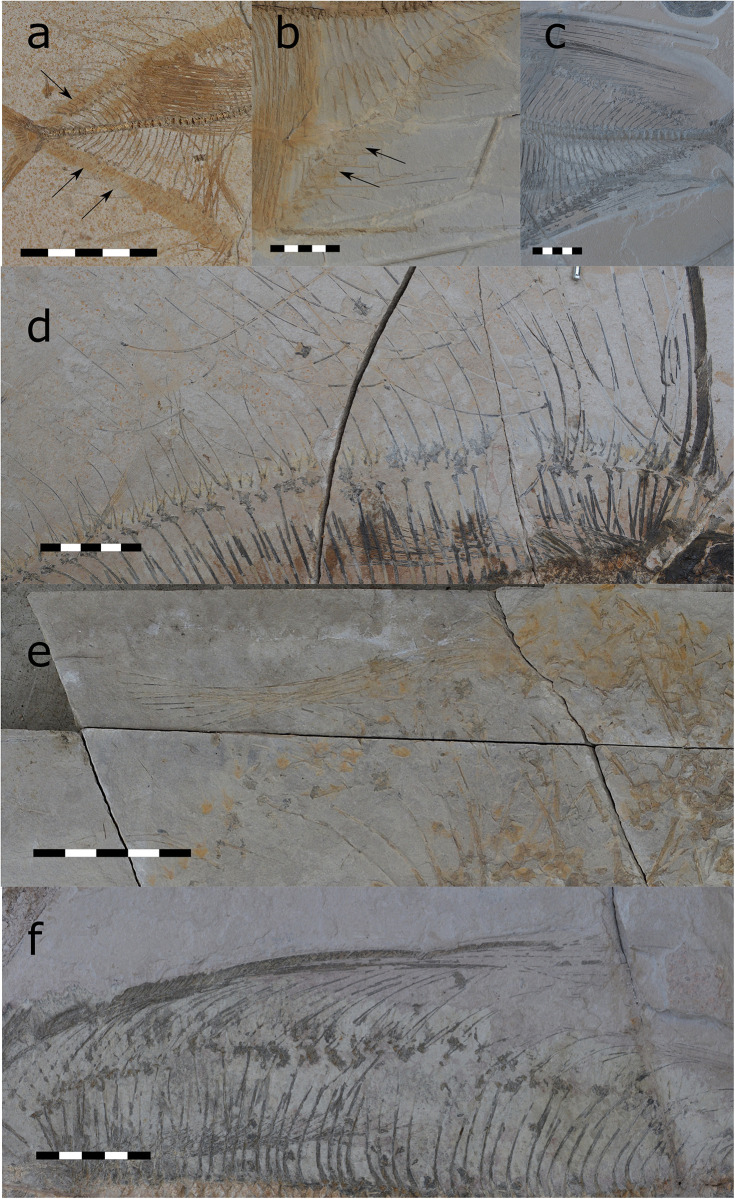
Preservation of the unpaired fins in *T*. *formosa*. A skin shadow (CPC-2832 and CPC-2883) (A and B) illustrates the sulcus to retract the dorsal and anal fin. Retracted dorsal fin without overlapping fin rays (CPC-2865) (C). Parallel arrangement of the dorsal fin rays (CPC-2960; REG2544 PF220) (D). Collective reaction of the fin rays similar to a fan indicating an intact membrane between the lepidotrichia (CPC-2902 and CPC-2871) (E and F). All scales 50 mm.

Giersch [9] and Taverne [38] suggested that *T*. *formosa* was able to fold back the dorsal and anal fin into a sulcus or groove. We agree with this interpretation. The strategy is comparable with that of modern fan fishes which retract the anal and dorsal fins behind modified scales at the base of the medial fins [39]. The sulcus of *T*. *formosa* is here built by lateral plates in which the fin can be retracted (Fig 14A and 14B). The middle segment sits between the pterygiophore and lepidotrichia, as already described by Giersch [9]. Each lepidotrichia sits on top of the middle segment, thus allowing for the retraction of the fin (Fig 14C). These lateral plates are here documented as skin shadow at the base of the rays (Fig 15A and 15B). The body shape therefore appears slightly expanded, similar to that in extant fan fishes.

Giersch argued that a fan of the dorsal and anal fin, such as seen in modern swordfishes, would be functionally problematic in *T*. *formosa* due to overlapping fin rays. Folded skin between individual lepidotrichia would then hamper the retraction of the unpaired fins and even make this movement impossible. However, specimens with retracted dorsal and anal fins present no evidence of damage of the fin rays (Fig 15C). Lepidotrichia do not overlap each other but remain parallel arranged (Fig 15D). The documented preservation of the unpaired fins therefore supports our assumption of a membrane between the fin rays. These show a consistent arrangement, even in disarticulated specimens. The fin rays thus behave in a connected way, similar to a fan. Delicate fin rays, as present in *T*. *formosa*, would easily have lost stability without a skin behaving as a stabilizer. Regarding the massive fin size the fin rays would easily detach, break, or would disintegrate in a chaotic way. Rays would then spread into different directions overlapping each other, which is not the case in the fossil specimens described here. On the contrary, it rather appears that fins were connected, bending collectively into one direction (e.g. as if exposed to a current) (Fig 15E and 15F). This leads us to the interpretation that the membrane was still intact. Furthermore, the impressive size of the dorsal and anal fins is not an argument to us to exclude a membrane, as extant fan fishes possess fins comparable to *T*. *formosa* [40–42]. We therefore suggest a similar retraction of the unpaired fins as seen in modern fan fishes which possess a membrane between the lepidotrichia.

Extant fan fishes (*Pteraclis* and *Pterycombus*) use their extremely large fins to avoid predators and to catch prey [39]. They have adapted methods to swim with high speed when retracting the fins and to improve their maneuverability by erecting them [39]. Considering the similarities of the huge dorsal and anal fin and the body shape of *T*. *formosa* we assume a similar strategy. Active hunting of prey thus seems likely in *T*. *formosa* based on its body outline.

## 5. Conclusion

A review of 149 individuals of *Tselfatia formosa* from the Lower to Middle Turonian platy limestone deposit of Vallecillo, northeastern Mexico, reveals the presence of two different body shape types which are interpreted to represent sexual shape dimorphism (SSD). Both types are present in small sized individuals interpreted as young and big sized specimens interpreted as adults. The unimodal size distribution of *T*. *formosa* illustrates one abundant peak between 500 and 600 mm length and a dominance of large and diamond-shaped individuals in the Vallecillo area. Diamond-shaped individuals are interpreted as deeply keeled females; they are twice as long as high compared to torpedo-shaped individuals interpreted as males with a length to width ratio of 3:1, thus being three times longer than wide. The abundance of well-articulated and complete specimens favors the rapid burial of carcasses, without an interference of scavengers and also excluding a phase of carcass flotation. The impressive dorsal and anal fins of *Tselfatia formosa* allow for comparison with modern fan fishes and a reconstruction of these fins with a membrane between each lepidotrichia. We therefore suggest that *T*. *formosa* inhabited a deep water environment and that its lifestyle resembled that of extant fan fishes.

## Supporting information

S1 File (PDF)
