# Supplementary material for: Taphonomy of the teleost Tselfatia formosa Arambourg, 1943 from Vallecillo, NE Mexico
Source: PLoS One. 2023 Feb 1;18(2):e0280797. doi: 10.1371/journal.pone.0280797 (PMC9891505; doi:10.1371/journal.pone.0280797)
Supplement: S1 File — (PDF) [file pone.0280797.s001.pdf]

Collection no	Specimen	locality
CPC-2821	*T. formosa*	Vallecillo
CPC-2822	*T. formosa*	Vallecillo
CPC-2947; REG2544 PF049	*T. formosa*	Vallecillo
CPC-2839	*T. formosa*	Vallecillo
CPC-2840	*T. formosa*	Vallecillo
CPC-2948; REG2544 PF48	*T. formosa*	Vallecillo
CPC-2949; REG2544 PF50	*T. formosa*	Vallecillo
CPC-2841	*T. formosa*	Vallecillo
CPC-2842	*T. formosa*	Vallecillo
CPC-2843	*T. formosa*	Vallecillo
CPC-2844	*T. formosa*	Vallecillo
CPC-2845	*T. formosa*	Vallecillo
CPC-2846	*T. formosa*	Vallecillo
CPC-2847	*T. formosa*	Vallecillo
CPC-2848	*T. formosa*	Vallecillo
CPC-2849	*T. formosa*	Vallecillo
CPC-2850	*T. formosa*	Vallecillo
CPC-2851	*T. formosa*	Vallecillo
CPC-2852	*T. formosa*	Vallecillo
CPC-2853	*T. formosa*	Vallecillo
CPC-2854	*T. formosa*	Vallecillo
CPC-2855	*T. formosa*	Vallecillo
CPC-2856	*T. formosa*	Vallecillo
CPC-2857	*T. formosa*	Vallecillo
CPC-2858	*T. formosa*	Vallecillo
CPC-2859	*T. formosa*	Vallecillo
CPC-2860	*T. formosa*	Vallecillo
CPC- 2861	*T. formosa*	Vallecillo
CPC- 2861	*T. formosa*	Vallecillo
CPC-2862	*T. formosa*	Vallecillo
CPC-2863	*T. formosa*	Vallecillo
CPC-2864	*T. formosa*	Vallecillo
CPC-2865	*T. formosa*	Vallecillo
CPC-2866	*T. formosa*	Vallecillo
CPC-2867	*T. formosa*	Vallecillo
CPC-2950; REG2544 PF218	*T. formosa*	Vallecillo
CPC-2868	*T. formosa*	Vallecillo
CPC-2951; REG2544 PF214	*T. formosa*	Vallecillo
CPC-2869	*T. formosa*	Vallecillo
CPC-2870	*T. formosa*	Vallecillo
CPC-2871	*T. formosa*	Vallecillo
CPC-2952; REG2544 PF228	*T. formosa*	Vallecillo

CPC-2953; REG2544 PF283	*T. formosa*	Vallecillo
CPC-2872	*T. formosa*	Vallecillo
CPC-2873	*T. formosa*	Vallecillo
CPC-2954; REG2544 PF280	*T. formosa*	Vallecillo
CPC-2874	*T. formosa*	Vallecillo
CPC-2955; REG2544 PF212	*T. formosa*	Vallecillo
CPC-2875	*T. formosa*	Vallecillo
CPC-2876	*T. formosa*	Vallecillo
CPC-2877	*T. formosa*	Vallecillo
CPC-2878	*T. formosa*	Vallecillo
CPC-2879	*T. formosa*	Vallecillo
CPC-2880	*T. formosa*	Vallecillo
CPC-2881	*T. formosa*	Vallecillo
CPC-2882	*T. formosa*	Vallecillo
CPC-2883	*T. formosa*	Vallecillo
CPC-2884	*T. formosa*	Vallecillo
CPC-2885	*T. formosa*	Vallecillo
CPC-2886	*T. formosa*	Vallecillo
CPC-2887	*T. formosa*	Vallecillo
CPC-2888	*T. formosa*	Vallecillo
CPC-2889	*T. formosa*	Vallecillo
CPC-2890	*T. formosa*	Vallecillo
CPC-2956; REG2544 PF213	*T. formosa*	Vallecillo
CPC-2957; REG2544 PF253	*T. formosa*	Vallecillo
CPC-2891	*T. formosa*	Vallecillo
CPC-2892	*T. formosa*	Vallecillo
CPC-2893	*T. formosa*	Vallecillo
CPC-2894	*T. formosa*	Vallecillo
CPC-2895	*T. formosa*	Vallecillo
CPC-2896	*T. formosa*	Vallecillo
CPC-2897	*T. formosa*	Vallecillo
CPC-2898	*T. formosa*	Vallecillo
CPC-2899	*T. formosa*	Vallecillo
CPC-2900	*T. formosa*	Vallecillo
CPC-2958; REG2544 PF168	*T. formosa*	Vallecillo
CPC-2901	*T. formosa*	Vallecillo
CPC-2959; REG2544 PF162	*T. formosa*	Vallecillo
CPC-2902	*T. formosa*	Vallecillo
CPC-2960; REG2544 PF220	*T. formosa*	Vallecillo
CPC-2903	*T. formosa*	Vallecillo
CPC-2904	*T. formosa*	Vallecillo
CPC-2905	*T. formosa*	Vallecillo
CPC-2906	*T. formosa*	Vallecillo

CPC-2907	*T. formosa*	Vallecillo
CPC-2908	*T. formosa*	Vallecillo
CPC-2909	*T. formosa*	Vallecillo
CPC-2910	*T. formosa*	Vallecillo
CPC-2911	*T. formosa*	Vallecillo
CPC-2912	*T. formosa*	Vallecillo
CPC-2913	*T. formosa*	Vallecillo
CPC-2914	*T. formosa*	Vallecillo
CPC-2915	*T. formosa*	Vallecillo
CPC-2916	*T. formosa*	Vallecillo
CPC-2917	*T. formosa*	Vallecillo
CPC-2918	*T. formosa*	Vallecillo
CPC-2919	*T. formosa*	Vallecillo
CPC-2961; REG2544 PF113	*T. formosa*	Vallecillo
CPC-2962; REG2544 PF151	*T. formosa*	Vallecillo
CPC-2920	*T. formosa*	Vallecillo
CPC-2921	*T. formosa*	Vallecillo
CPC-2922	*T. formosa*	Vallecillo
CPC-2923	*T. formosa*	Vallecillo
CPC-2924	*T. formosa*	Vallecillo
CPC-2963; REG2544 PF219	*T. formosa*	Vallecillo
CPC-2964; REG2544 PF062	*T. formosa*	Vallecillo
CPC-2925	*T. formosa*	Vallecillo
CPC-2926	*T. formosa*	Vallecillo
CPC-2927	*T. formosa*	Vallecillo
CPC-2928	*T. formosa*	Vallecillo
CPC-2929	*T. formosa*	Vallecillo
CPC-2930	*T. formosa*	Vallecillo
CPC-2931	*T. formosa*	Vallecillo
CPC-2965; REG2544 PF211	*T. formosa*	Vallecillo
CPC-2966; REG2544 PF208	*T. formosa*	Vallecillo
CPC-2967; REG2544 PF275	*T. formosa*	Vallecillo
CPC-2968; REG2544 PF205	*T. formosa*	Vallecillo
CPC-2823	*T. formosa*	Vallecillo
CPC-2824	*T. formosa*	Vallecillo
CPC-2825	*T. formosa*	Vallecillo
CPC-534	*T. formosa*	Vallecillo
CPC-2826	*T. formosa*	Vallecillo
CPC-2827	*T. formosa*	Vallecillo
CPC-2828	*T. formosa*	Vallecillo
CPC-2829	*T. formosa*	Vallecillo
CPC-987	*T. formosa*	Vallecillo
CPC-2830	*T. formosa*	Vallecillo

CPC-2831	*T. formosa*	Vallecillo
CPC-2832	*T. formosa*	Vallecillo
CPC-2833	*T. formosa*	Vallecillo
CPC-2969	*T. formosa*	Vallecillo
CPC-2970	*T. formosa*	Vallecillo
CPC-2834	*T. formosa*	Vallecillo
CPC-2932	*T. formosa*	Vallecillo
CPC-2933	*T. formosa*	Vallecillo
CPC-2934	*T. formosa*	Vallecillo
CPC-2935	*T. formosa*	Vallecillo
CPC-2936	*T. formosa*	Vallecillo
CPC-2937	*T. formosa*	Vallecillo
CPC-2938	*T. formosa*	Vallecillo
CPC-2939	*T. formosa*	Vallecillo
CPC-2940	*T. formosa*	Vallecillo
CPC-2941	*T. formosa*	Vallecillo
CPC-2942	*T. formosa*	Vallecillo
CPC-2943	*T. formosa*	Vallecillo
CPC-2944	*T. formosa*	Vallecillo
CPC-2945	*T. formosa*	Vallecillo
CPC-2946	*T. formosa*	Vallecillo
CPC-2835	*T. formosa*	Mesa las Tabla
CPC-2836	*Dixonanogmius sp.*	San Carlos
CPC-2837	*Dixonanogmius sp.*	Temporales
CPC-2838	*Dixonanogmius sp.*	San Carlos

CPC-MUSEO DEL DESIERTO
Colección Paleontológica de Coahuila
REG-Instituto Nacional de Antropología e Historia
